# Correction: Effect of ionic size compensation by Ag^+^ incorporation in homogeneous Fe-substituted ZnO: studies on structural, mechanical, optical, and magnetic properties

**DOI:** 10.1039/c8ra90069h

**Published:** 2018-08-16

**Authors:** Gaurav Bajpai, Tulika Srivastava, N. Patra, Igamcha Moirangthem, S. N. Jha, D. Bhattacharyya, Sk Riyajuddin, Kaushik Ghosh, Dharma R. Basaula, Mahmud Khan, Shun-Wei Liu, Sajal Biring, Somaditya Sen

**Affiliations:** Metallurgical Engg. and Material Sciences, Indian Institute of Technology Indore India sens@iiti.ac.in; Department of Physics, Indian Institute of Technology Indore India; Atomic & Molecular Physics Division, Bhabha Atomic Research Centre Mumbai India; Amity Institute of Nanotechnology Noida U.P. India; Institute of Nano Science and Technology Mohali Punjab India; Department of Physics, Miami University Oxford Ohio 45056 USA; Electronic Engg., Ming Chi University of Technology New Taipei City Taiwan biring@mail.mcut.edu.tw

## Abstract

Correction for ‘Effect of ionic size compensation by Ag^+^ incorporation in homogeneous Fe-substituted ZnO: studies on structural, mechanical, optical, and magnetic properties’ by Gaurav Bajpai *et al.*, *RSC Adv.*, 2018, **8**, 24355–24369.

The authors regret that incorrect insets were shown in Fig. 1(b) and (d) in the original manuscript. The corrected figure is shown below.

**Fig. 1:**
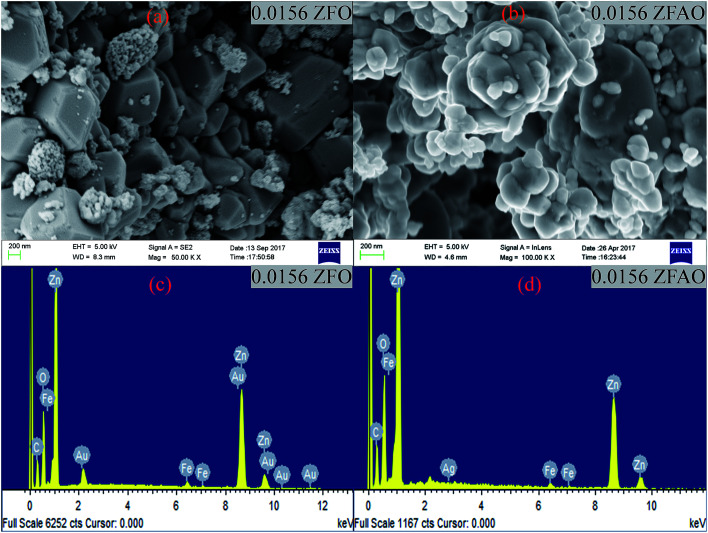
(a) and (b) FESEM images of 0.0156 ZFO & 0.0156 ZFAO, (c) and (d) EDX of 0.0156 ZFO & 0.0156 ZFAO.

In addition, on page 24359 in the sentence “Pre-edge peak arises due to intermixing of Fe/Zn 3d and O 4p states”, “O 4p” should be replaced by “4p”. The correct sentence is as follows:

“Pre-edge peak arises due to intermixing of Fe/Zn 3d and 4p states”.

The Royal Society of Chemistry apologises for these errors and any consequent inconvenience to authors and readers.

## Supplementary Material

